# Treatment outcomes of tuberculosis patients under directly observed treatment short-course at Debre Tabor General Hospital, northwest Ethiopia: nine-years retrospective study

**DOI:** 10.1186/s40249-018-0395-6

**Published:** 2018-02-26

**Authors:** Seble Worku, Awoke Derbie, Daniel Mekonnen, Fantahun Biadglegne

**Affiliations:** 1 Department of Medical Laboratory Sciences, College of Health Sciences, Debre Tabor University, Debre Tabor, Ethiopia; 2 Department of Medical Microbiology, Immunology and Parasitology, College of Medicine and Health Sciences, Bahir Dar University, P.O. Box 1383, Bahir Dar, Ethiopia; 3 Biotechnology Research Institute, Bahir Dar University, Bahir Dar, Ethiopia

**Keywords:** Tuberculosis, Treatment outcome, DOTS, Debre Tabor General Hospital, Ethiopia

## Abstract

**Background:**

Data regarding tuberculosis (TB) treatment outcomes, proportion of TB/HIV co-infection and associated factors have been released at different TB treatment facilities in Ethiopia and elsewhere in the world as part of the auditing and surveillance service. However, these data are missing for the TB clinic offering directly observed treatment short-course (DOTs) at Debre Tabor General Hospital (DTGH).

**Methods:**

The authors analysed the records of 985 TB patients registered at the DTGH from September 2008 to December 2016. Data on patients’ sex, age, type of TB, and treatment outcomes were extracted from the TB treatment registration logbook. The treatment outcome of patients was categorized according to the National TB and Leprosy Control Program guidelines: cured, treatment completed, treatment failed, died, and not evaluated (transferred out and unknown cases).

**Results:**

Around half of the registered patients were males (516, 52.4%). In terms of TB types, 381 (38.7%), 241 (24.5%), and 363 (36.9%) patients had smear-negative pulmonary TB, smear-positive pulmonary TB, and extra pulmonary TB, respectively. Six hundred and seventy-two patients (90.1%) had successful treatment outcomes (cured and treatment completed), while 74 patients (9.9%) had unsuccessful treatment outcomes (death and treatment failure).TB treatment outcome was not associated with age, sex, type and history of TB, or co-infection with HIV (*P* > 0.05). The proportion of TB/HIV co-infection was at 24.2%, and these were found to be significantly associated with the age groups of 25–34, 35–44 and ≥65 years:(a*OR*: 0.44; 95% *CI*: 0.25–0.8), (a*OR*: 0.39; 95% *CI*: 0.20–0.70), (*aOR*: 4.2; 95% *CI*: 1.30–12.9), respectively.

**Conclusions:**

The proportion of patients with successful treatment outcomes was above the World Health Organization target set for Millennium Development Goal of 85% and in line with that of the global milestone target set at > 90% for 2025. Relatively higher proportions of transfer-out cases were recorded in the present study. Similarly, the proportion of TB/HIV co-infection cases was much higher than the national average of 8%.Thus, the health facility under study should develop strategies to record the final treatment outcome of transfer-out cases. In addition, strategies to reduce the burden of TB/HIV co-infection should be strengthened.

**Electronic supplementary material:**

The online version of this article (10.1186/s40249-018-0395-6) contains supplementary material, which is available to authorized users.

## Multilingual abstracts

Please see Additional file 1 for translations of the abstract into the five official working languages of the United Nations.

## Background

Tuberculosis (TB) is one of the most serious public health challenges worldwide. Globally, around 10.4 million people develop TB and 1.8 million people die from it (0.4 million of these also have HIV) [1].

China and India account for more than half of the TB burden worldwide [1]. Sub-Saharan Africa, including Ethiopia, has the highest prevalence of TB infection in the world. In 2015, a World Health Organization (WHO) report showed that Ethiopia ranked seventh out of 22 countries with the highest TB burden [1].

The WHO has implemented the standardized directly observed treatment, short-course (DOTS)/Stop TB Strategy to scale up TB prevention and control. The TB control program in Ethiopia introduced health facility-based DOTS as a pilot program in 1992 [2] and at the Debre Tabor General Hospital (DTGH) in 2000.The geographic coverage of the DOTS strategy in the country is 71% [1]. The TB treatment success rate for all forms of TB in 2014 was 89%, which was close to the global milestone target set at > 90% for 2025 [1].

Even though the objectives of TB treatment are curing patients, as well as preventing the spread of TB infection and the emergence of new drug-resistant strains, these are not achieved in many regions of the world due to several factors. These include: the severity of the disease, co-infection with HIV and/or other diseases, multidrug resistance, poverty and the support provided to patients such as; helping them to take their TB medications regularly and to complete TB treatment as well as financial, social and psychological support [3–9].

Treatment outcomes of TB have been evaluated at some of the facilities providing DOTS in Ethiopia [3–9]. In these studies, the age, sex, and residence of patients, as well as the form of TB have been reported to affect treatment outcome. Investigating factors affecting treatment outcomes of TB patients helps to improve the performance of DOTS and informs TB infection and control programs.

In Ethiopia, which is characterized by poor surveillance system, as well as poor supportive supervision and mentorship for health professionals, possible recognition and amendment of system failures are unlikely [observation]. Although a number of DOTS experiences have been reported in Ethiopia [3–9], treatment outcomes of patients and associated factors have not been assessed in the present study area. Moreover, the TB/HIV co-infection rate and its association with TB treatment outcomes have not been assessed at the TB clinic of the DTGH as part of the auditing service. Thus, this study was conducted to assess TB treatment outcomes and their associated factors at this facility.

## Materials and methods

### Study setting

This study was conducted at the DTGH. The hospital is a general healthcare-level setting that serves general medical service for the population of Debre Tabor town and its surroundings. The total population served by the hospital is about five million. TB patients may go to the hospital with or without referral.

The DOTS clinic at the hospital operates under the National TB and Leprosy Program (NTLCP) of Ethiopia. Patients used to be diagnosed for TB using the spot-morning-spot smear strategy, but now they are diagnosed using the spot-spot strategy and chest radiographs [10].

To test for TB microscopically, sputum smears are prepared, air dried, fixed, stained using the Ziehl-Neelsen method, and examined by direct microscopy for acid-fast bacilli (AFB) using oil-immersion (100 ×) objectives, according to the NTLCP protocol [2]. If the sputum smear is negative, doctors establish the diagnosis of TB primarily based on clinical grounds such as chest X-ray. Patients who test positive are referred to the DOTS clinic where they are registered and treated, as according to the NTLCP.

### Study design and data collection

A nine-year retrospective descriptive analysis to assess treatment outcomes and associated risk factors pertaining to the 985 registered TB patients was carried out at the DOTS clinic at the DTGH from September 2008 to December 2016. Patients were provided with free TB medications for a period of 6–9 months. All 985 patients were followed up during their course of treatment to assess treatment outcomes. Demographic data such as patients’ age, sex, place of residence, clinical data, HIV sero-status, TB type, and treatment outcomes were captured in the registration form.

Patients’ treatment outcomes were evaluated according to the NTLCP and classified as either: cured (finished treatment with negative bacteriology result at the end of treatment); treatment completed (finished treatment but without bacteriology result at the end of treatment); defaulted (patients who interrupted their treatment for two consecutive months or more after registration); treatment failure (remaining smear-positive at five months despite correct intake of medication); died (patients who died from any cause during the course of treatment); transferred out (patients whose treatment result is unknown due to transfer to another health facility); successfully treated (a patient who was cured or completed treatment); and unsuccessfully treated (patients whose treatment was interrupted, who were transferred out or who failed treatment) [2, 11, 12].

### Statistical analysis

All data were entered, cleared, and analysed using the SPSS Statistics software package version 22.0 (IBM SPSS Statistics for Windows, Armonk, NY: IBM Corp.). Descriptive data analysis such as proportion, median, and range were used to visualize differences within the data. Moreover, a stepwise logistic regression model was constructed to assess factors associated with TB treatment outcomes in terms of an odds ratio (*OR*) and its 95% confidence interval (*CI*). The *P*-value was set at 0.05 to indicate a statistically significant difference. In the univariate analysis, variables with *P*-values of < 0.2 were subject to a multivariate analysis.

## Results

In this study, data pertaining to 985 TB patients were reviewed. Slightly more than half of the total registered patients were male (516, 52.4%) with a median age of 28 years. About a third of the patients were in the age group of 15–24 years (292, 29.6%) and about a quarter were in the age group of 25–34 years (235, 23.9%). The majority of TB patients were newly diagnosed (832, 84.5%). Of the total TB patients, 381 (38.7%), 363 (36.9%), and 241 (24.5%) were smear-negative pulmonary tuberculosis (PTB-), extra pulmonary tuberculosis (EPTB), and smear-positive pulmonary tuberculosis (PTB+) cases, respectively. The proportion of TB/HIV co-infection cases was 238 (24.2%) (see Table 1).

**Table 1:** Demographic and TB-related history of patients at the DTGH, 2008–2016

Variable		Frequency	Percentage
Sex	Male	516	52.4
	Female	469	47.6
Age group (in years)	0–14	99	10.1
	15–24	292	29.6
	25–34	235	23.9
	35–44	151	15.3
	45–54	84	8.5
	55–64	54	5.5
	≥ 65	70	7.1
TB registration status	New	832	84.5
	Relapsed	57	5.8
	Failed	18	1.8
	Transfer in	70	7.1
	Other	8	0.8
Type of TB	PTB+	241	24.5
	PTB-	381	38.7
	EPTB	363	36.9
HIV status	Positive	238	24.2
	Negative	706	71.7
	Unknown	41	4.2
Total		985	100.0

**Fig. 1:**
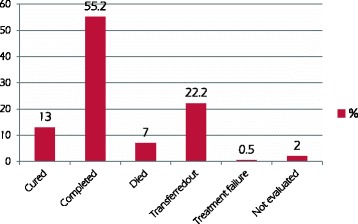
Proportion of TB treatment outcome of the study participants (*n* = 985), Debre Tabor General Hospital, 2016

Excluding the transferred out (*n* = 219) and unknown treatment outcome (*n* = 20) cases, there were 672 (90.1%) patients with successful treatment outcomes (cured and treatment completed), while 74 patients (9.9%) had unsuccessful treatment outcomes (death and treatment failure) (see Fig. 1). Among the HIV-positive TB patients included in the analysis for TB treatment outcome (*n* = 176), the treatment success rate was 88.1%, only about 2% lower than the success treatment rate of all subjects.

Taking into account the TB patients with known treatment outcomes, a regression analysis was conducted to assess whether treatment outcomes were associated with patients’ sex, age, HIV status, and type and history of TB. No statistically significant differences among these variables against the TB treatment outcome were found (see Table 2).

**Table 2:** Logistic regression analysis showing associations between TB treatment outcomes and potential predictors, DTGH, 2008–2016

Variables		TB treatment outcome^a^		c*OR* (95% *CI*)	*P* value	a*OR* (95% *CI)*	*P* value
		Unsuccessful *n* (%)	Successful *n* (%)				
Sex	Male	45 (5.7)	344 (94.3)	1		1	
	Female	29 (8.1)	328 (91.9)	1.5(0.92.4)	0.12	1.5(0.9–2.5)	0.10
Age group (in years)	0–14	8 (11.6)	61(88.4)	1		1	
	15–24	17 (7.2)	220(92.)	1.7(0.7–4)	0.24	1.8(0.73–4.46)	0.20
	25–34	11 (6.4)	160(93.)	1.9(0.7–5)	0.19	2(0.77–5.4)	0.15
	35–44	18 (16.5)	91 (83.5)	0.66(0.27–1.6)	0.37	0.73(0.29–1.8)	0.50
	45–54	7 (9.2)	60 (90.8)	1.12(0.38–3.3)	0.8	1.18(0.4–3.5)	0.76
	55–64	6 (8.3)	36 (91.7)	0.78(0.25–2.45)	0.68	0.8(0.25–2.5)	0.70
	≥ 65	7 (13.7)	44 (86.3)	0.8(0.28–2.4)	0.72	0.86(0.3–2.6)	0.78
Type of TB	PTB+	18 (9.5)	171(90.5)	1		1	
	PTB-	29 (10.5)	247(89.5)	0.89(0.48–1.66)	0.70	1.04(0.5–2)	0.89
	EPTB	27 (9.6)	254(90.4)	0.99(0.5–1.8)	0.98	1.04(0.5–2)	0.89
TB registration status	New	62 (10.1)	550(89.9)	1		1	
	Relapse	4 (8.2)	45 (91.8)	1.3(0.44–3.6)	0.66	1.48(0.5–4.4)	0.47
	Treatment after Failure	1(5.9)	16 (94.1)	1.8(0.23–31.8)	0.57	1.6(0.2–12.7)	0.65
	Transfer in	6 (9.7)	56 (90.3)	1.05(0.44–2.5)	0.9	0.93(0.37–3)	0.87
	Other	1 (16.7)	5 (83.3)	0.56(0.06–4.9)	0.6	0.37(0.04–3.3)	0.37
HIV status	Positive	21 (11.9)	155(88.1)	1		1	
	Negative	50 (9.2)	494(90.8)	1.33(0.78–2.3)	0.30	1.37(0.76–2.4)	0.23
	Unknon	3 (11.5)	23 (88.5)	1.03(0.28–3.76)	0.95	1.13(0.3–4.2)	0.5

^a^NB: the calculation excluded transferred out (*n* = 219) and treatment outcome unknown (20) cases

Analysis was also done to assess whether TB/HIV co-infection was associated with patients’ sex, age, and types of TB. The odds of having HIV was higher by 44% (a*OR*: 0.44; 95% *CI*: 0.25–0.8) and 39% (a*OR*: 0.39; 95% *CI*: 0.20–0.70) among those patients in the age groups of 25–34 and 35–44 years, respectively, when compared with those in the age group of 0–14 years. On the contrary, those in the age group of ≥65 years were 4.2 times more likely to have a TB/HIV co-infection than children (*aOR*: 4.2; 95% *CI*:1.30–12.9) (see Table 3).

**Table 3:** Regression analysis showing the association between TB/HIV co-infection cases and patients’ sex, age and types of TB, at the DTGH, 2008–2016

		TB/HIV co-infection		*cOR (95% CI)*	*P* value	*aOR (95% CI)*	*P* value
		Yes, *n* (%)	No, *n* (%)				
Sex	Male	117 (23.9)	373 (76.1)	1		1	
	Female	121 (26.7)	333 (73.3)	0.9 (0.60–1.20)	0.33	0.92 (0.68–1.20)	0.62
Types of TB	PTB+	67 (28.8)	166 (71.2)	1		1	
	PTB-	95 (26.0)	270 (74.0)	1.12 (0.8–1.70)	0.46	1(0.7–1.50)	0.88
	EPTB	76 (22.0)	270 (78.0)	1.40 (1.0–2.10)	0.06	1.4(0.92–2.0)	0.12
Age group (in years)	0–14	19 (19.8)	77 (80.2)	1		1	
	15–24	49 (17.3)	235 (82.7)	1.2 (0.66–2.13)	0.60	1.2(0.66–2.0)	0.56
	25–34	81 (36.3)	142 (63.7)	0.43 (0.24–0.77)	0.004	0.44(0.25–0.80)	0.006
	35–44	57 (39.3)	88 (60.7)	0.38 (0.21–0.70)	0.002	0.39(0.20–0.70)	0.002
	44–54	22 (27.8)	57 (72.2)	0.64 (0.32–1.30)	0.21	0.67(0.33–1.40)	0.27
	55–64	6 (8.9)	43 (91.9)	1.8(0.66–4.80)	0.30	1.8(0.67–4.80)	0.25
	≥ 65	4 (5.9)	64 (94.1)	3.9(1.30–12.20)	0.012	4.2 (1.30–12.9)	0.014

## Discussion

Tuberculosis remains a major public health problem around the globe, especially in developing nations like Ethiopia. Assessing TB treatment outcomes is important for stakeholders working to evaluate the performance of TB treatment strategies such as DOTS and patient-related factors.

Our present and previous studies [13, 14], and other similar studies [6, 15] conducted in Ethiopia, consistently show that TB presence is relatively higher among males than females. So far, the reports of consistently high prevalence rates of TB among the male population were discussed poorly in Ethiopia. However, emerging reports outside of Ethiopia show host genetic bases for such differences [16–19]. Furthermore, sexual hormones, sex-related genetic background and genetic regulations, and metabolism, among other factors, might contribute in susceptibility differences between men and women [17, 20]. Recently, an *X* chromosome susceptibility gene has been suggested as explaining the higher susceptibility of males than females to TB [17].

In this study, the burden of TB was higher among the age groups of 15–24 and 25–34 years. This is in line with many similar studies that confirm as TB mainly affects sexually active and reproductive age groups. This might also be due to high HIV prevalence in these age groups [12].

The proportion of PTB+, PTB-, and EPTB cases were24.5%, 38.7%, and 36.9%, respectively. The proportion of PTB+ cases was relatively lower when compared to the national average reported by the WHO between 2011 and 2015, of 32.4% [21–24]. Moreover, bacteriologically confirmed TB is also much lower than the national average of 54% [1]. However, there are similar reports showing lower notification rates of PTB+ cases and high numbers of EPTB cases in the northern part Ethiopia [3, 13–15, 25, 26]. The causes for this are not yet identified and some reports suggest that it might have something to do with the host genomic base, but this needs further investigation [27]. On top of this, due to limited resources, most of the health facilities in Ethiopia have been using AFB microscopy, which has low sensitivity (40–45%) and might contribute to lower notification rates of PTB+ (observation). The results of this study could be used to support the country’s strategy to use rapid, sensitive, and specific point-of-care diagnostic technologies such as GeneXpert among vulnerable groups (children, HIV positive people, etc.).

The proportion of TB/HIV co-infection cases was 24.2%, which is much higher than the national prevalence of 8% [1]. This could partly be explained by the fact that the subjects in the present study were individuals with active TB, who might have undying immunological defect like HIV. On top of this, the national TB/HIV co-infection estimate was based on the community-based survey. At the same time, our findings are from one site, while the national data is countrywide and thus disparity is likely. However, similar findings to the present study have also been reported by other studies conducted in Ethiopia [28–30].

In the present study, the proportion of patients with successful treatment outcomes (cured and completed) was found to be 90.9%, which is higher than the WHO target set for the Millennium Development Goal(MDG) of 85% [31–33] and comparable to that of the milestone target set globally for 2025 of > 90% [1, 34].Comparable findings were reported by other Ethiopian studies [5, 26, 35–37].

In this study, large proportions of TB patients were transferred out of the DTGH. This result is in line with our previous report [14]. This might be due to these studies being conducted in hospitals that have been serving a wider population. Thus, once these patients are diagnosed, they are transferred to a nearby health facility for follow-up. When comparing hospitals with lower level health facilities such as health centres, it is found that hospitals have a higher proportion of transfer-out cases [25]. Due to this, we have excluded transfer-out and unknown treatment outcome cases in the subgroup analysis for tracing associated factors with treatment outcome.

Among the HIV-positive TB patients, the treatment success achieved was 88.1%, only about 2% lower than the success treatment rate of all subjects. The 2% unsuccessful treatment outcome among HIV co-infected TB patients might be attributed to factors such as underlying HIV and other undiagnosed opportunistic infections.

Tuberculosis treatment outcome was not associated with patients’ age, sex, type and history of TB, and TB/HIV co-infection (*P* > 0.05) (see Table 2). This might be due to the statistical pooling effect of the reference groups. In contrast to our study, other studies have shown an association between HIV co-infection and TB treatment outcome [27, 35].

Though not statistically significant, this study showed that the proportion of patients who were successfully treated for TB/HIV co-infection was higher among female PTB+ patients (see Table 3). This is in line with a similar report from the Tigray region, Ethiopia [36] and might be related with the lesser probability of females engaging in risky behaviours such as alcohol, substance, and tobacco abuse (observation). However, TB/HIV co-infection was significantly associated with the age groups of 25–34 and 35–44 years (*aOR*: 0.44; 95% *CI*: 0.25–0.8) and (a*OR*: 0.39; 95% *CI*: 0.2–0.7), respectively. A similar finding was reported by another study [38]. This might be explained by the higher prevalence of HIV among these age groups, which could increase the chance of reactivation of HIV-associated TB. On top of this, participants in the age group of ≥65 years were found 4.2 times more likely to have a TB/HIV co-infection than children (*aOR*: 4.2; 95% *CI*: 1.30–12.9) that might be due to their declined immune status.

The results of our study should be applied with caution when evaluating the overall TB treatment success rate in the studied region. Our findings are subject to at least three limitations. The first is selection bias. The second is that the study site (DTGH) is a general hospital, in which patients could come from other health facilities to be diagnosed. Third, patients were first admitted to the DTGH and after starting the intensive phase of treatment, they transferred out to their nearby health facilities. Thus, their final treatment outcomes (cured, failure, default, or death) were not captured. At the same, due to the retrospective nature of the study, it was not possible to show detailed clinical profiles of the TB patients, which might play a significant role in indicating the overall picture of the study participants.

Nevertheless, our study tried to provide baseline information about treatment outcomes of TB patients and showed related key gaps, such as the large amount of undocumented treatment outcome data. The results of this study also indicate that TB is still a major public health problem at the studied area.

## Conclusions

This study showed that TB mainly affected individuals in the reproductive age group. The proportion of patients who were successfully treated was above the MDG target of 85% treatment success rate and comparable with the milestone target of > 90% treatment success rate.

A relatively higher number of transfer-out cases were recorded in this study. Thus, the studied health facility should network with other health service centres to document the treatment outcomes of patients.

The proportion of TB/HIV co-infection was 24%, which was much higher than the national average of 8%. Thus, early HIV and TB screening and antiretroviral treatment initiation should be put in place. Moreover, the use of isoniazid prophylaxis should be maximized to prevent HIV-associated TB.

## Additional file

Additional file 1: Multilingual abstract in the five official working languages of the United Nations. (PDF 512 kb)

## Abbreviations

AFB: Acid-fast bacilli
*CI*: Confidence interval
DOTS: Directly observed treatment, short-course
DTGH: Debre Tabor General Hospital
EPTB: Extra pulmonary tuberculosis
HIV: Human immunodeficiency virus
MDG: Millennium Development Goal
NTLC: National TB and Leprosy Program
PTB**-**: Smear-negative pulmonary tuberculosis
PTB **+**: Smear-positive pulmonary tuberculosis
TB: Tuberculosis
WHO: World Health Organization

## References

[1] World health organization (2016). Global tuberculosis Report 2016.

[2] Cfdcapo E, Federal Democratic Republic of Ethiopia MoH (2012). Guidelines for clinical and programmatic Management of Tb. Leprosy and Tb/Hiv in Ethiopia.

[3] Tessema B, Muche A, Bekele A, Reissig D, Emmrich F, Sack U (2009). Treatment outcome of tuberculosis patients at Gondar University teaching hospital, Northwest Ethiopia. A five-year retrospective study. BMC Public Health.

[4] Tadesse S, Tadesse T (2014). Treatment success rate of tuberculosis patients in Dabat, northwest Ethiopia. Health.

[5] Berhe G, Enquselassie F, Aseffa A (2012). Treatment outcome of smear-positive pulmonary tuberculosis patients in Tigray region, northern Ethiopia. BMC Public Health.

[6] Muñoz-Sellart M, Cuevas L, Tumato M, Merid Y, Yassin M (2010). Factors associated with poor tuberculosis treatment outcome in the southern region of Ethiopia. Int J Tuberc Lung Dis.

[7] Getahun B, Ameni G, Medhin G, Biadgilign S (2013). Treatment outcome of tuberculosis patients under directly observed treatment in Addis Ababa, Ethiopia. Braz J Infect Dis.

[8] Woldeyohannes D, Kebede N, Erku W, Tadesse Z (2011). Ten years experience of directly observed treatment short-course (dots) therapy for tuberculosis in Addis Ababa. Ethiopia Ethiop Med J.

[9] Esmael A, Tsegaye G, Wubie M, Abera H, Endris M (2014). Treatment outcomes of tuberculosis patients in Debre Markos referral hospital, north West Ethiopia (June 2008-august 2013): a five year retrospective study. Int J Pharm Sci Res.

[10] Tesfaye A, Fiseha D, Assefa D, Klinkenberg E, Balanco S, Langley I (2017). Modeling the patient and health system impacts of alternative xpert® MTB/RIF algorithms for the diagnosis of pulmonary tuberculosis in Addis Ababa, Ethiopia. BMC Infect Dis.

[11] World Health Organization (2006). Guidance for national tuberculosis programmes on the management of tuberculosis in children.

[12] World Health Organization (2015). Definitions and reporting framework for tuberculosis 2013 revision [updated December 2014].

[13] Mekonnen D, Derbie A, Mekonnen H, Zenebe Y (2016). Profile and treatment outcomes of patients with tuberculosis in northeastern Ethiopia: a cross sectional study. Afr Health Sci.

[14] Zenebe Y, Adem Y, Mekonnen D, Derbie A, Bereded F, Bantie M (2016). Profile of tuberculosis and its response to anti-TB drugs among tuberculosis patients treated under the TB control programme at Felege-Hiwot referral hospital, Ethiopia. BMC Public Health.

[15] Gebrezgabiher G, Romha G, Ejeta E, Asebe G, Zemene E, Ameni G (2016). Treatment outcome of tuberculosis patients under directly observed treatment short course and factors affecting outcome in southern Ethiopia: a five-year retrospective study. PLoS One.

[16] Klein SL (2000). The effects of hormones on sex differences in infection: from genes to behavior. Neurosci Biobehav Rev.

[17] Neyrolles O, Quintana-Murci L (2009). Sexual inequality in tuberculosis. PLoS Med.

[18] Liu W, Cao W, Zhang C, Tian L, Wu X, Habbema J (2004). VDR and NRAMP1 gene polymorphisms in susceptibility to pulmonary tuberculosis among the Chinese Han population: a case-control study. Int J Tuberc Lung Dis.

[19] Sørensen TI, Nielsen GG, Andersen PK, Teasdale TW (1988). Genetic and environmental influences on premature death in adult adoptees. N Engl J Med.

[20] Möller M, Hoal EG (2010). Current findings, challenges and novel approaches in human genetic susceptibility to tuberculosis. Tuberculosis.

[21] World Health Organization. Global tuberculosis report. 2013. https://reliefweb.int/sites/reliefweb.int/files/resources/9789241564656_eng.pdf. Accessed 27 Jan 2017.

[22] World Health Organization. Global tuberculosis control. WHO report. 2011:2011. https://www.scribd.com/document/68374316/Global-Tuberculosis-Control-2011. Accessed 27 Jan 2017

[23] World Health Organization. Global tuberculosis report 2012. https://reliefweb.int/sites/reliefweb.int/files/resources/9789241564502_eng.pdf. Accessed 27 Jan 2017.

[24] World Health Organization. Global tuberculosis report 2015. WHO/HTM/TB/2015.22. Geneva, WHO Press; 2015.

[25] Endris M, Moges F, Belyhun Y, Woldehana E, Esmael A, Unakal C. Treatment outcome of tuberculosis patients at Enfraz health center, Northwest Ethiopia: a five-year retrospective study. Tuberc Res Treat. 2014;2014:1–7. https://www.hindawi.com/journals/trt/2014/726193/. 10.1155/2014/726193 PMC4027020 24891948

[26] Ejeta E, Chala M, Arega G, Ayalsew K, Tesfaye L, Birhanu T (2015). Outcome of tuberculosis patients under directly observed short course treatment in western Ethiopia. J Infect Dev Ctries.

[27] Berg S, Schelling E, Hailu E, Firdessa R, Gumi B, Erenso G (2015). Investigation of the high rates of extrapulmonary tuberculosis in Ethiopia reveals no single driving factor and minimal evidence for zoonotic transmission of Mycobacterium bovis infection. BMC Infec Dis.

[28] Mekonnen D, Derbie A, Desalegn ETB (2015). HIV co-infections and associated factors among patients on directly observed treatment short course in northeastern Ethiopia: a 4 years retrospective study. BMC Res Notes.

[29] Denegetu AW, Dolamo BL (2014). HIV screening among TB patients and co-trimoxazole preventive therapy for TB/HIV patients in Addis Ababa: facility based descriptive study. PLoS One.

[30] Ali SA, Mavundla TR, Fantu R, Awoke T (2016). Outcomes of TB treatment in HIV co-infected TB patients in Ethiopia: a cross-sectional analytic study. BMC Infec Dis..

[31] Dye C, Maher D, Weil D, Espinal M, Raviglione M (2006). Targets for global tuberculosis control. Int J Tuberc Lung Dis.

[32] Raviglione MC, Uplekar MW (2006). WHO’s new stop TB strategy. Lancet.

[33] World Health Organization. The Stop TB Strategy: Building on and enhancing DOTS to meet the TB-related Millennium Development Goals. 2006. http://www.eldis.org/document/A24279. Accessed 27 Jan 2017.

[34] Uplekar M, Weil D, Lonnroth K, Jaramillo E, Lienhardt C, Dias HM (2015). WHO's new end TB strategy. Lancet.

[35] Gebremariam G, Asmamaw G, Hussen M, Hailemariam MZ, Asegu D, Astatkie A (2016). Impact of HIV status on treatment outcome of tuberculosis patients registered at Arsi Negele health center, southern Ethiopia: a six year retrospective study. PLoS One.

[36] Belayneh M, Giday K, Lemma H (2015). Treatment outcome of human immunodeficiency virus and tuberculosis co-infected patients in public hospitals of eastern and southern zone of Tigray region, Ethiopia. Braz J Infect Dis.

[37] Amante TD, Ahemed TA (2015). Risk factors for unsuccessful tuberculosis treatment outcome (failure, default and death) in public health institutions. Eastern Ethiopia Pan Afr Med J.

[38] Gesesew H, Tsehaineh B, Massa D, Tesfay A, Kahsay H, Mwanri L (2016). The role of social determinants on tuberculosis/HIV co-infection mortality in southwest Ethiopia: a retrospective cohort study. BMC Res Notes.

