# Supplementary material for: Treatment outcomes of tuberculosis patients under directly observed treatment short-course at Debre Tabor General Hospital, northwest Ethiopia: nine-years retrospective study
Source: Infect Dis Poverty. 2018 Feb 26;7:16. doi: 10.1186/s40249-018-0395-6 (PMC6389154; doi:10.1186/s40249-018-0395-6)
Supplement: Supplementary file 1 — Multilingual abstract in the five official working languages of the United Nations. (PDF 512 kb) [file 40249_2018_395_MOESM1_ESM.pdf]

Translation of the abstract into the five official working languages of the United Nations

**نتائج علاج مرضى السل ضمن برنامج مباشر الرقابة قصير الأمد في مستشفى دبري تبر الحكومي القابع شمال شرق أثيوبيا: دراسة استعادية لتسعة سنوات**

سبلي وركو، أوكيدربي، دنيل مكنن، فتهون بيرفلني.

**الموجز**

**التمهيد:** تمّ نشر المعلومات بشأن نتائج علاج داء السل، ونسب الإصابة بهذا الداء وبنقص المناعة البشرية معاً، وعوامل الخطر المرتبطة بها في مختلف مرافق علاج داء السل في أثيوبيا وفي شتى أنحاء العالم كجزء من جهود القائمين على مراقبة وتدقيق الخدمة، ولكن مستشفى دبري تبر الحكومي لا يزال يفتقر لمثل هذه المعلومات عن العلاج مباشر الرقابة قصير الأمد لداء السل.

**المنهجية:** قمنا بدراسة سجلات 985 من مرضى السل المسجلين في المستشفى بين تاريخ سبتمبر 2008 و ديسمبر 2016، وتمّ حصد معلومات حول جنس المريض وعمره، ونوع داء السل الذي يعاني منه، ونتائج العلاج الذي خضع له من دفتر السجلات، وقد تمّ تصنيف نتائج علاج المرضى بما يتماشى مع تعليمات البرنامج الوطني لمكافحة السل والجذام، والتي توضح إذا ما كان المريض قد: تماثل للشفاء، أو أتمّ فترة علاجه، أو لم يستفد من العلاج، أو توفي، أو غير مصنف (إما بسبب نقل المريض من المستشفى أو لأن هوية المريض غير معروفة).

**النتائج:** كان نحو نصف عدد المرضى المسجلين في المستشفى ذكوراً (516- 52.4%)، أما من ناحية نوع داء السل فقد تبين أن 381 مريضاً (38.7%) مصابٌ بالدرن الرئوي السلبي، و 241 مريضاً (24.5%) مصابٌ بالدرن الرئوي الإيجابي، و363 مريضاً (36.9%) مصابٌ بالسل خارج الرئة. وقد نجح علاج 672 مريضاً (90.1%) (تماثلوا للشفاء بعد إتمامهم لفترة العلاج)، في حين قد بائت محاولات علاج 74 مريضا (9.9%) بالفشل (أي أنّ المرضى قد توفوا أو أن العلاج لم يكن فعالاً معهم)، ولم يكن نجاح أو فشل العلاج مرتبطاً بجنس المريض وعمره، أو نوع داء السل الذي يعاني أو قد سبق وعانى منه، ولكن اتضح أن هنالك ارتباطاً واضحاً عند أولائك المصابين بداء السل وبنقص المناعة البشرية في الفئتين العمريتين 25-34 [aOR: 0.44; (95%CI: (0.25 – 0.8)]، و 35-44 [a*OR*: 0.39; 95% *CI*: (0.2 – 0.7)].

**الاستنتاجات:** كانت نسب نجاح علاج داء السل أعلى من الهدف الذي وضعته منظمة الصحة العالمية ضمن هدف الإنماء للألفية (85%)، وتتماشى مع النسب التي تطمح المنظمة لتحقيقها في عام 2025 (<90%). هذا وتم في هذا البحث رصد عدد أكبر من حالات أولائك الذين نقلوا سجلاتهم من المستشفى، كما تبين أنّ نسب المصابين بكلّ من داء السل ونقص المناعة البشرية معاً أعلى من المتوسط الوطني البالغ 8%، مما يعني أنّ على المرافق الصحية المعنية تطوير إستراتيجيات تمكنها من تسجيل نتائج العلاج النهائية للمرضى المنقولين، والعمل على تحسين إستراتيجيات تقلل من نسب الإصابة بداء السل ونقص المناعة البشرية معاً.

Translated from English version into Arabic by Alaa AlHalabi, through

**埃塞俄比亚西北部德卜勒塔博尔总医院结核病患者直接督导短程化疗治疗结果：9年的回顾性研究**

Seble Worku, Awoke Derbie, Daniel Mekonnen and Fantahun Biadglegne

**摘要：**

**引言：**作为审计和监督服务的一部分，关于结核病(TB)的治疗结果、结核病与艾滋病合并感染比例以及相关风险因素的数据已经在埃塞俄比亚和世界其他地方不同的结核治疗机构发布。然而，德卜勒塔博尔总医院（DTGH）结核科提供的直接督导短程化疗（DOTs）结果缺少这些数据。

**方法：**我们分析了 2008 年 9 月至 2016 年 12 月在 DTGH 登记的 985 例结核病患者记录。从结核病治疗记录中获得患者性别、年龄、结核分型和治疗结果的数据。根据国家结核病和麻风病防控规划指南对患者治疗结果进行分类：治愈、治疗完成、治疗失败、死亡、未评估（转出和未知病例）。

**结果：**约一半的登记患者为男性（516，52.4％）。在结核分型方面，381 例（38.7％）、241 例（24.5％）和 363 例（36.9％）患者分别为痰涂片阴性肺结核、痰涂片阳性肺结核和肺外结核。672 例患者（90.1％）治疗成功（包括治愈和治疗完成），74 例（9.9％）治疗不成功（包括死亡和治疗失败）。结核病治疗结果与患者年龄、性别、分型、病史，或合并 HIV 感染无关（$P > 0.05$）。238 例（24.2％）为结核和艾滋合并感染病例，这与 25-34 岁[a*OR*：0.44; 95％*CI*：（0.25-0.8）]、35-44 岁[a*OR*：0.39; 95％*CI*：（0.2-0.7）]年龄组具有显著相关性。

**结论：**治疗成功的患者比例高于世界卫生组织千年发展目标设定的 85％，与全球里程碑目标设定的 2025 年达到 90％以上相当。本研究中病例转出比例相对较高。同样地，结核和艾滋合并感染病例的比例远高于 8％的全国平均水平。因此，本研究中的卫生机构应制定策略，记录转出病例的最终治疗结果。此外，还应加强对策从而减轻结核和艾滋合并感染负担。

Translated from English version into Chinese by Peng Song, edited by Pin Yang

CHINESE CENTER FOR DISEASE CONTROL AND PREVENTION
NATIONAL INSTITUTE OF PARASITIC DISEASES

**Résultats de traitement des patients tuberculeux sous traitement de courte durée sous observation directe à l’Hôpital général de Debre Tabor, dans le nord-ouest de l’Éthiopie : étude rétrospective sur neuf ans**

Seble Worku, Awoke Derbie, Daniel Mekonnen et Fantahun Biadglegne

**Résumé**

**Contexte :** Des données concernant les résultats de traitement de la tuberculose, la proportion de co-infections par la tuberculose et le VIH et les facteurs de risque associés ont été publiées dans différents centres de traitement antituberculeux, en Éthiopie et ailleurs dans le monde, aux fins d'audit et de surveillance. Il n'existe pas de telles données pour le service de l'Hôpital général de Debre Tabor (DTGH) proposant des traitements de courte durée sous observation directe (DOT).
**Méthodes :** Nous avons analysé les dossiers de 985 patients tuberculeux enregistrés au DTGH entre septembre 2008 et décembre 2016. Des données sur le sexe et l'âge des patients, le type de tuberculose et le traitement ont été extraites des registres de traitement de la tuberculose. Le résultat du traitement a été catégorisé selon les instructions du Programme national de lutte contre la tuberculose et la lèpre : guérison, traitement terminé, échec du traitement, décès, non évalué (transféré ou perdu de vue).
**Résultats :** La moitié environ des patients enregistrés étaient de sexe masculin (516, 52,4 %). La tuberculose était pulmonaire à frottis négatif chez 381 patients (38,7 %), pulmonaire à frottis positif chez 241 (24,5 %) et extrapulmonaire chez 363 (36,9 %). Le traitement a donné de bons résultats (guérison ou traitement terminé) chez 672 patients (90,1 %) mais un résultat négatif (décès ou échec du traitement) chez 74 autres (9,9 %). L'issue du traitement de la tuberculose n'avait aucun lien avec l'âge, le sexe, le type ni l'historique de la tuberculose, ni avec la co-infection par le VIH ($P > 0,05$). La proportion de co-infections tuberculose-VIH était de 238 patients (24,2 %), significativement associés aux groupes d'âge de 25 à 34 ans (ORa : 0,44 ; IC à 95 % de 0,25 à 0,8) et de 35 à 44 ans (ORa : 0,39 ; IC à 95 % de 0,2 à 0,7).
**Conclusions :** La proportion de patients traités avec succès était supérieure à l'objectif de 85 % fixé par l'Organisation mondiale de la Santé dans ses Objectifs du Millénaire pour le développement et conforme à l'objectif de jalon global fixé à > 90 % pour 2025. Une proportion relativement élevée de cas transférés a été relevée dans la présente étude. La proportion des co-infections tuberculose-VIH était également beaucoup plus élevée que la moyenne nationale de 8 %. Le centre de santé étudié devrait donc élaborer des stratégies pour enregistrer l'issue du traitement des cas transférés. Les stratégies visant à réduire le poids des co-infections tuberculose-VIH devraient également être renforcées.

Translated from English version into French by Suzanne Assenat, through

TRANSLATORS
WITHOUT BORDERS

**Результаты краткосрочного лечения больных туберкулезом при непосредственном наблюдении в многопрофильной больнице Дебре Табор, Северо-Западная Эфиопия: Ретроспективное исследование в течение девяти лет**

Себле Ворку, Авоке Дербие, Даниэль Меконнен и Фантахун Биадглегне

**Аннотация**

**Данные:** В различных лечебных учреждениях в Эфиопии, и других районах мира в рамках ревизии и наблюдения были опубликованы данные об итогах лечения туберкулеза, о доле сочетанного инфицирования туберкулезом/ВИЧ и сопутствующих факторах риска. Однако в этих данных отсутствует информация по туберкулезной клинике, осуществляющей непосредственное кратковременное наблюдение в многопрофильной больнице Дебре Табор (ДТ).

**Методы:** Мы проанализировали истории болезни 985 больных туберкулезом, зарегистрированных в (ДТ) с сентября 2008 по декабрь 2016. Данные о половых сношениях пациентов, возрасте, типах туберкулеза и лечении были получены из регистрационного журнала. Результаты лечения пациентов были классифицированы в соответствии с национальными руководством по борьбе с туберкулезом и проказой: излеченные, с завершенным лечением, с незавершенным лечением, умершие и не зарегистрированные (перемещенные и неизвестные случаи).

**Результаты:** Около половины зарегистрированных пациентов составляли мужчины (516 - 52.4%).С точки зрения типов туберкулеза, 381 (38,7%), 241 (24,5%), и 363 (36,9%) пациентов имели легочную форму туберкулеза (ТБ) с отрицательными результатами микроскопии мазка мокроты, легочную форму ТБ с положительными результатами микроскопии мазка мокроты и внелегочную форму ТБ соответственно. 672 пациента (90,1 %) имели успешные результаты лечения (вылечились и завершенное лечение), в то время как 74 пациента (9,9%) имели неудачные результаты лечения (смерть и незавершенное лечение). Результаты лечения туберкулеза не связаны с возрастом, полом, типом и историей туберкулеза, а также с коинфекцией ВИЧ ($P > 0,05$).Доля случаев сочетанного инфицирования туберкулезом/ВИЧ составляла 238 (24,2%), и эти случаи были преимущественно в возрастных группах 25-34 и 35-44 лет:[a*OR*:0.44; 95%*CI*:(0.25 – 0.8)] и [a*OR*:0.39; 95% *CI*:(0.2 – 0.7)], соответственно.

**Заключение:** Доля пациентов, добившихся успешных результатов лечения была выше целевого показателя Всемирной организации здравоохранения, сформулированного в Декларации тысячелетия в 85%, но соответствовала глобальным целевым показателем >90% на 2025 год. В настоящем исследовании были отмечены относительно более высокие пропорции случаев перемещенных дел. Аналогичным образом, доля случаев сочетанного инфицирования туберкулезом и ВИЧ значительно выше, чем в среднем по стране на 8%. Таким образом, в исследуемом медицинском учреждении следует разработать стратегии регистрации окончательных результатов обработки перемещенных дел. Кроме того, следует усилить стратегии профилактики сочетанного инфицирования туберкулезом и ВИЧ.

Translated from English version into Russian by TatianaKary, through

TRANSLATORS
WITHOUT BORDERS

**Resultados del tratamiento de los pacientes con tuberculosis sometidos a tratamiento corto bajo observación directa en el Hospital General Debre Tabor, al noroeste de Etiopía: estudio retrospectivo de nueve años.**

Seble Worku, Awoke Derbie, Daniel Mekonnen y Fantahun Biadglegne

**Resumen**

**Antecedentes:**
Los datos relativos a los resultados del tratamiento de la tuberculosis (TB), la proporción de coinfección TB/VIH y los factores de riesgo asociados se han divulgado en diferentes instalaciones de tratamiento de la tuberculosis en Etiopía y en otras partes del mundo como parte del servicio de auditoría y seguimiento. Sin embargo, faltan estos datos para la clínica de TB que ofrece tratamiento de observación directa a corto plazo (DOT) en el Hospital General Debre Tabor (DTGH).
**Métodos:**
Se analizaron los registros de 985 pacientes con TB registrados en el DTGH desde septiembre de 2008 hasta diciembre de 2016. Los datos sobre el sexo, la edad, el tipo de TB y los resultados del tratamiento de los pacientes se extrajeron del registro del tratamiento de la TB. El resultado del tratamiento de los pacientes se clasificó según las directrices del Programa Nacional de Control de la Tuberculosis y la Lepra: curado, tratamiento terminado, tratamiento frustrado, muerto y no evaluado (casos transferidos y desconocidos).
**Resultados:**
Alrededor de la mitad de los pacientes registrados eran varones (516/52,4%). En cuanto a los tipos de TB: 381 (38,7%), 241 (24,5%) y 363 (36,9%) pacientes tuvieron TB pulmonar con frotis negativo, TB pulmonar con frotis positivo y TB pulmonar adicional, respectivamente. Seiscientos setenta y dos pacientes (90,1%) tuvieron resultados de tratamiento exitosos (curado y tratamiento terminado), mientras que 74 pacientes (9,9%) tuvieron resultados de tratamiento sin éxito (muerte y fracaso del tratamiento). El resultado del tratamiento de la tuberculosis no se asoció con la edad, el sexo, el tipo y el historial de tuberculosis o la coinfección con el VIH ($P > 0,05$). La proporción de casos de coinfección TB/VIH fue de 238 (24,2%), y se encontró que se asociaron significativamente con los grupos de edad de 25 a 34 y 35 a 44 años: [aOR: 0,44; IC del 95%: (0,25 a 0,8)] y [aOR: 0,39; IC del 95%: (0,2 a 0,7)], respectivamente.
**Conclusiones:**
La proporción de pacientes con resultados de tratamiento satisfactorios fue superior a la meta fijada por la Organización Mundial de la Salud para el Objetivo de Desarrollo del Milenio del 85% y en consonancia con el objetivo de la meta histórica mundial de > 90% para 2025. En el presente estudio se registraron proporciones relativamente mayores de casos de traslado. De manera similar, la proporción de casos de coinfección TB/VIH fue mucho mayor que la media nacional del 8%. Por lo tanto, el establecimiento de salud bajo estudio debe desarrollar estrategias para registrar el resultado final del tratamiento de los casos transferidos. Además, deben reforzarse las estrategias para reducir la carga de la coinfección TB/VIH.

Translated from English version into Spanish by patclivio, through

TRANSLATORS
WITHOUT BORDERS
